# WHO Basic Emergency Care (BEC) training in Liberia: pre-course, post-course, and one-year outcomes

**DOI:** 10.1016/j.afjem.2026.101019

**Published:** 2026-10-22

**Authors:** Omeed Miraftab-Salo, Ibrahim Ajami, Nathan Mahteh, Alexa Lucas, Naomi Faulk, Michele Ricks-Dargba, Tabehde Freeman-Murray, Michael Jaung, Corey Bills, Kayla Enriquez

**Affiliations:** a University of California, San Francisco Department of Emergency Medicine, USA; b 14 Military Hospital, Liberia Ministry of Health & African Federation of Emergency Medicine, Republic of Liberia; c Redemption Hospital, Liberia Ministry of Health & African Federation of Emergency Medicine, Republic of Liberia; d Baylor College of Medicine, Henry JN Taub Department of Emergency Medicine, Houston, TX, USA; e University of Colorado Anschutz School of Medicine, Department of Emergency Medicine, USA

**Keywords:** Emergency medicine, Basic emergency care, Liberia, Medical education, Knowledge retention, Global health

## Abstract

**Introduction:**

Liberia faces persistent gaps in emergency care training. The World Health Organization (WHO) Basic Emergency Care (BEC) Course was developed in 2018 to support training of frontline emergency care providers in resource-limited settings. While prior studies have shown short-term knowledge gains and confidence improvements, limited data exists regarding longer-term retention. This study sought to evaluate knowledge acquisition, knowledge retention, and self-reported confidence immediately after and one year following WHO Basic Emergency Care training.

**Methods:**

We conducted two four-day WHO BEC trainings for graduating medical students and practicing providers at A.M. Dogliotti School of Medicine in Monrovia, Liberia. Participants completed knowledge tests, self-assessment surveys, and course evaluations before training, immediately after training, and at one-year follow-up. Paired pre/post comparisons were analyzed using Wilcoxon signed-rank tests, and cohort comparisons for post-course practical case scenarios and course evaluations used Fisher’s exact test and unpaired t-tests, respectively.

**Results:**

Some 40 participants completed training, including 30 medical students, nine nurses, and one general practitioner. 33 of 40 participants (82.5%) completed the one-year follow-up assessment. Median knowledge scores increased from 76% pre-course to 96% immediately post-course and remained higher than baseline at one year (88%; all *p* < 0.0001). Self-reported confidence also improved substantially, including comfort managing emergency patients and preparedness to see emergency care patients, with gains sustained at one year. Improvements were observed across key emergency care domains, including shock, airway management, pediatric and adult emergencies, and emergency pharmacology. Course evaluations were highly positive across all domains, with mean ratings ranging from 3.8 to 5.0 out of 5.

**Discussion:**

WHO BEC training was associated with substantial improvements in emergency care knowledge and confidence among Liberian providers, with evidence of knowledge retention at one year. These findings support implementation of WHO BEC training as a sustainable strategy to strengthen emergency care capacity in resource-limited African settings.

## African Relevance

• WHO Basic Emergency Care (BEC) course is a feasible approach to strengthening emergency care capacity in African health systems.
• Emergency care training can improve knowledge and self-reported confidence among frontline healthcare providers in Liberia.
• Educational gains following WHO BEC training were sustained at one year, supporting the durability of emergency care education.
• Integrating WHO BEC into undergraduate and continuing education may strengthen emergency care systems across Africa.
• These findings support investment in scalable emergency care training programs to improve the care in African settings.

## Introduction

A substantial proportion of preventable morbidity and mortality in low- and middle-income countries (LMICs) is attributable to acute, time-sensitive conditions [1]. Estimates from the Disease Control Priorities project suggest that over half of deaths in LMICs could be addressed through timely emergency and essential surgical care [2]. Despite this burden, emergency care systems in many LMICs remain under-resourced, and access to timely, high-quality emergency care is limited. More specifically, in Liberia, 52.7% of the Disability Adjusted Life Years and 65.7% of Years of Life Lost (YLL) are due to lack of acute care. Moreover, Liberia held the lowest percentage of YLL attributable to chronic disease of all countries in the 2019 Global Burden of Disease study at 59% compared to 98% in the US, highlighting the immense acute care burden of disease in the country [3].

In these settings, emergency care is frequently delivered by healthcare providers without specialist emergency medicine training, including generalist physicians and nurses with variable formal training in acute care [4]. Strengthening early recognition, triage, and stabilization through targeted education is therefore a critical strategy for improving patient outcomes [5]. Structured training programs that focus on foundational emergency care principles have been proposed as scalable approaches to address these gaps.

The World Health Organization jointly developed the Basic Emergency Care (BEC) course in 2018 with partners to address gaps in emergency care training for frontline providers working in resource-limited settings. The BEC course emphasizes a syndromic, systems-based approach and has been implemented across diverse global contexts [6]. Prior evaluations have demonstrated short-term improvements in participant knowledge and confidence following course completion [7]. However, longer-term knowledge retention or sustained clinical impact remains poorly understood [8].

The objective of this study was to evaluate knowledge retention, self-reported confidence, and perceived clinical competence among healthcare providers participating in a longitudinal emergency care training program. By assessing outcomes immediately following training and at one-year follow-up, this study aims to inform the design of sustainable emergency care training models in other resource-limited settings.

## Methods

This was a quasi-experimental educational intervention study with pre-course, immediate post-course, and one-year assessments to evaluate knowledge acquisition and retention. All participants received the WHO BEC intervention; there was no untreated control group. Participants comprised two healthcare provider cohorts: final-year medical students and practicing providers (nine nurses and one general practitioner). Participant knowledge, attitudes, and perceptions about the concepts and skills taught were assessed before and after course implementation.

This study was reviewed by the University of California, San Francisco Institutional Review Board and certified as exempt (IRB #25-43364) and the University of Liberia Institutional Review Board, Atlantic Center for Research and Evaluation (ACRE) (Protocol #25-03-455). All participants provided oral informed consent prior to participation. The use of oral consent in lieu of written consent was approved by both institutional review boards. Participation was voluntary, and all study data were de-identified prior to analysis.

Participant recruitment and training were conducted in March 2024 at A.M. Dogliotti School of Medicine in Monrovia, Liberia. Thirty fifth-year medical students from A.M. Dogliotti School of Medicine, representing the entire graduating class for that year, participated in the study. Nine nurses and one doctor from 14 Military Hospital who had not previously taken the BEC course and were selected by the BEC master trainer, also participated. No formal sample size calculation was performed because all eligible final-year medical students and selected practicing providers participating in the training were invited to participate.

Eligible participants were adult healthcare providers, including final-year medical students at A.M. Dogliotti School of Medicine and practicing nurses or physicians selected for the provider cohort. Exclusion criteria included non-consent, age younger than 18 years, incarceration, pregnancy, or disability. No consenting participants met exclusion criteria. The practicing-provider cohort had not previously completed the WHO BEC course. Prior BLS training or employment in an emergency department or intensive care setting were not eligibility criteria.

A.M Dogliotti School of Medicine provided the venue and day of support for the two 4-day trainings and 1-day Training of Trainers, as well as classroom materials and AV support. The African Federation for Emergency Medicine (AFEM) regional office in conjunction with the World Health Organization (WHO) regional office provided BEC materials and workbooks for participants as well as recommendation for national Master and Provisional Trainers. The AFEM Master Trainer assisted in getting approval from the Liberia Ministry of Health to host the course, worked with A.M. Dogliotti School of Medicine to schedule the course and enroll 5th year medical students and recruited 3 Provisional Trainers to teach the course.

The 40 participants were divided into two cohorts of 15 medical students and five 14 Military Hospital staff per cohort. Each cohort of 20 participants completed the four-day long World Health Organization Basic Emergency Care (BEC) package training. Each training was taught by three BEC provisional trainers and one BEC master trainer, yielding a participant-to-trainer ratio of 5:1. Two trainers were from the United States, and the remaining trainers were Liberian physicians and surgeons.

The WHO BEC course is distinct from Basic Life Support (BLS) training and provides broader instruction in the initial assessment and stabilization of acutely ill and injured patients. The four-day curriculum incorporated didactic lectures, small-group sessions, and practical skills training. Course content included the ABCDE approach, clinical handover, difficulty in breathing, primary and secondary trauma assessment, shock, altered mental status, and emergency medications. Practical training included three 1.5 h skills sessions focused on airway and breathing interventions, circulation interventions, and neurological assessment, immobilization, and wound care. Hands-on skills training was augmented using Laerdal torso and head manikins to practice basic airway management, cervical spine immobilization, sucking chest wound management, needle decompression, bleeding control, and fracture stabilization. On the final day, participants completed an additional 1 h and 40 min practical assessment session that included the Practical Case Scenario (PraCS) assessment.

After completion of each four-day training, five participants from each cohort (10 total) were selected to participate in a one-day WHO BEC Training of Trainers session. A summary of the four-day BEC training schedule is provided in Supplementary Material 1.

Participants completed standardized WHO BEC assessment instruments at predefined study timepoints. Knowledge was assessed using the 25-item WHO BEC multiple-choice examination, which evaluates core emergency care domains including the ABCDE approach: altered mental status, difficulty in breathing, shock, and trauma. A distinct post-course examination was administered immediately after training, and knowledge was reassessed at one-year follow-up. Self-reported confidence and perceived competence were assessed pre-course, post-course, and at one year using the WHO BEC Self-Efficacy Survey. This instrument assesses general confidence and preparedness for emergency care as well as confidence across specific clinical domains, including care of acutely ill and injured adults and children, shock, altered mental status, difficulty in breathing, emergency medications, airway and breathing skills, bleeding management, and immobilization.

Practical skills were assessed immediately post-course using the WHO BEC Practical Case Scenario (PraCS) assessment, which evaluates identification and performance of critical airway, breathing, circulation, disability, exposure, and medication interventions and completion of an appropriate clinical handover. Course feedback was collected immediately post-course using the WHO BEC Participant Course Feedback instrument, which assesses the appropriateness, clarity, and quality of course content and educational materials, instructor effectiveness, and participant perceptions of the course.

Pre- and immediate post-course knowledge assessments were administered in person under classroom-style proctoring with BEC facilitators and trainers present, and practical skills assessments were also proctored by trained BEC personnel. The one-year assessment was administered electronically and remotely and was not proctored. No additional measures to monitor use of external resources during the one-year assessment were implemented. No refresher training or additional BEC instruction was provided between the immediate post-course and one-year assessments. Practical skills were assessed only immediately post-course and were not reassessed at one year.

The primary outcome for this study was knowledge gain after course completion defined by improvement in pre- and post-test scores and knowledge retention defined by post test score comparison immediately following completion of the course to one-year after completion of the course.

Secondary outcomes were changes in attitude and perception around course content and participant confidence with knowledge and skills attained.

Statistical analyses were performed using STATA Version 17 (StataCorp, College Station, TX, USA). Continuous variables were summarized using medians and interquartile ranges. Paired comparisons between pre-course, post-course, and one-year follow-up scores were performed using the Wilcoxon signed-rank test. Categorical variables were compared using chi-square or Fisher’s exact tests as appropriate. A two-sided *p*-value <0.05 was considered statistically significant. Analyses were conducted using complete paired data available at each assessment time point; no imputation of missing data was performed.

## Results

Forty participants completed the BEC training, including 30 medical students, nine nurses, and one general practitioner. Participant characteristics are shown in Table 1. All medical students in the final year of training at A.M. Dogliotti School of Medicine were invited to participate, and no consenting participants were ineligible.

**Table 1:** Demographic characteristics of participants by cohort.

Characteristic	Total (*N* = 40)	Medical Student Cohort (*N* = 30)	Military 14 Cohort (*N* = 10)
*Sex,n(%)*			
Female	26 (65)	17 (57)	9 (90)
Male	14 (35)	13 (43)	1 (10)
*Highestleveloftraining,n(%)*			
Medical school	31 (77.5)	30 (100)	1 (10)
Nursing	9 (22.5)	0 (0)	9 (90)
*Yearsworkinginhealthcare,n(%)*			
0	30 (75)	30 (100)	0 (0)
1–5	7 (17.5)	0 (0)	7 (70)
>5	3 (7.5)	0 (0)	3 (30)

Values are presented as n (%).

MS = Medical Student.

Mil 14 = Military Hospital Cohort.

Median test scores significantly increased from pre-test to post-test across all participants (76% to 96%, *p* < 0.0001), with medical students increasing from 76% to 96% (*p* < 0.0001) and 14 Military Hospital practicing providers (nurses and general practitioner) increasing from 60% to 88% (*p* = 0.0058). At one year, the total cohort’s performance remained above the pre-test baseline of 76% for all participants with a median of 88%, *p* < 0.001, with medical students increasing from 76% to 88%, *p* < 0.001. Practicing providers had a nonsignificant increase in score from 60% to 76%, *p* = 0.2436 (see Table 2).

**Table 2:** Knowledge test scores before, immediately after, and one year after the BEC course.

Cohort	Pretest Median (IQR)	Post-test Median (IQR)	1-year Median (IQR)	*p*-value (pre–post)	*p*-value (post–1 year)	*p*-value (pre–1 year)
Total	0.76 (0.68–0.84)	0.96 (0.92–1.00)	0.88 (0.80–0.92)	<0.0001	<0.0001	<0.0001
Medical students	0.76 (0.68–0.84)	0.96 (0.96–1.00)	0.88 (0.84–0.92)	<0.0001	<0.0001	<0.0001
Military staff	0.60 (0.56–0.72)	0.88 (0.84–0.92)	0.76 (0.68–0.88)	0.0058	0.026	0.2436

*P*-values calculated using Wilcoxon signed-rank tests among participants with paired data at each timepoint. One-year follow-up data were available for 33 participants. IQR = Interquartile Range.

Self-reported confidence and perceived skill increased substantially following training and remained higher at one year across most domains. For example, comfort managing emergency patients increased from 1.58 to 3.30, and preparedness to see emergency care patients increased from 1.73 to 3.48. Gains were also observed in confidence with very ill patients, understanding the ABCDE approach, and emergency care skills related to shock, airway management, dyspnea, and immobilization. Small declines at one year were noted in some domains, including airway-related skills and emergency drugs, but scores remained above baseline overall (Table 3, Table 4).

**Table 3:** Self-assessed confidence before, immediately after, and one year after BEC training.

	Military Staff (*N* = 10)		Medical Students (*N* = 30)		Total Participants (*n* = 40)		Total 1-year Respondents (*n* = 33)	
**Survey Item**	Pre-BEC Mean (SD)	Post-BEC Mean (SD)	Pre-BEC Mean (SD)	Post-BEC Mean (SD)	Pre-BEC Mean (SD)	Post-BEC Mean (SD)	Post-BEC Mean (SD)	1-year Mean (SD)
Comfortable managing emergency patients	2.30 (1.42)	3.00 (1.15)	1.33 (0.99)	3.40 (0.50)	1.58 (1.17)	3.30 (0.72)	3.32 (0.77)	3.36 (0.49)
Nervous about emergencies	1.70 (1.42)	0.90 (1.20)	2.53 (1.33)	0.83 (1.09)	2.33 (1.38)	0.85 (1.10)	0.85 (1.18)	0.85 (0.61)
Others have emergency skills	3.00 (1.41)	3.30 (0.95)	2.43 (1.17)	3.00 (1.02)	2.58 (1.24)	3.08 (1.00)	3.06 (1.07)	2.97 (0.64)
Lack skills in emergencies	0.60 (0.52)	0.60 (1.26)	2.43 (1.25)	0.43 (0.82)	1.98 (1.37)	0.48 (0.93)	0.44 (0.99)	0.50 (0.51)
Prepared for emergencies	3.30 (0.48)	3.30 (1.25)	1.20 (1.00)	3.53 (0.51)	1.73 (1.28)	3.48 (0.75)	3.53 (0.79)	3.36 (0.49)
Confident with very ill patients	3.20 (1.23)	3.40 (0.97)	1.47 (1.07)	3.33 (0.55)	1.90 (1.34)	3.35 (0.66)	3.50 (0.51)	3.03 (0.80)
Uncomfortable with protocols	0.70 (0.48)	0.80 (1.23)	1.43 (1.28)	0.93 (1.53)	1.25 (1.17)	0.90 (1.45)	0.85 (1.46)	0.79 (1.14)
Understand ABCDE approach	2.70 (0.95)	3.70 (0.48)	2.43 (1.33)	3.97 (0.18)	2.50 (1.24)	3.90 (0.30)	3.91 (0.29)	3.65 (0.49)
Organized emergency approach	2.30 (1.16)	3.50 (0.53)	1.63 (1.19)	3.77 (0.43)	1.80 (1.20)	3.70 (0.46)	3.76 (0.43)	3.32 (0.53)
Not confident in knowledge	1.10 (0.88)	0.00 (0.00)	2.27 (1.17)	0.20 (0.41)	1.98 (1.21)	0.15 (0.36)	0.18 (0.39)	0.56 (0.50)
Training is important	3.80 (0.42)	3.90 (0.32)	3.97 (0.18)	3.97 (0.18)	3.93 (0.27)	3.95 (0.22)	3.97 (0.17)	3.65 (0.77)

Values are presented as mean (standard deviation). Likert scale responses ranged from 0 to 4, with higher values indicating greater confidence or skill. One-year follow-up data were available for 33 participants (24 medical students and 9 practicing providers). Results are presented descriptively.

Military Cohort (practicing providers: 9 nurses and 1 physician).

**Table 4:** Self-assessed emergency care skills before, immediately after, and one year after BEC training.

	Military Staff (*N* = 10)		Medical Students (*N* = 30)		Total Participants (*n* = 40)		Total 1-year Respondents (*n* = 33)	
**Survey Item**	Pre-BEC Mean (SD)	Post-BEC Mean (SD)	Pre-BEC Mean (SD)	Post-BEC Mean (SD)	Pre-BEC Mean (SD)	Post-BEC Mean (SD)	Post-BEC Mean (SD)	1-year Mean (SD)
Acutely ill adult	1.50 (0.71)	2.50 (0.71)	0.73 (0.58)	2.43 (0.50)	0.93 (0.69)	2.45 (0.55)	2.53 (0.56)	2.35 (0.54)
Acutely ill child	1.20 (0.63)	2.30 (0.67)	0.57 (0.50)	2.40 (0.56)	0.73 (0.60)	2.38 (0.59)	2.44 (0.56)	2.15 (0.50)
Injured adult	1.90 (0.57)	2.60 (0.52)	0.87 (0.68)	2.47 (0.51)	1.13 (0.79)	2.50 (0.51)	2.59 (0.50)	2.41 (0.50)
Injured child	1.70 (0.48)	2.30 (0.48)	0.70 (0.65)	2.50 (0.51)	0.95 (0.74)	2.45 (0.50)	2.53 (0.51)	2.27 (0.45)
Shock	1.40 (0.52)	2.40 (0.84)	0.87 (0.51)	2.57 (0.50)	1.00 (0.55)	2.53 (0.60)	2.59 (0.56)	2.26 (0.51)
Altered mental status	0.70 (0.67)	2.30 (0.82)	0.70 (0.53)	2.40 (0.50)	0.70 (0.56)	2.38 (0.59)	2.41 (0.56)	2.03 (0.59)
Dyspnea	1.60 (0.52)	2.70 (0.48)	0.80 (0.66)	2.47 (0.51)	1.00 (0.72)	2.53 (0.51)	2.56 (0.50)	2.35 (0.49)
Emergency drugs	1.60 (0.97)	2.20 (0.63)	0.33 (0.55)	2.13 (0.63)	0.65 (0.86)	2.15 (0.62)	2.21 (0.59)	1.88 (0.54)
Airway skills	1.40 (0.84)	2.50 (0.53)	0.50 (0.63)	2.53 (0.51)	0.73 (0.78)	2.53 (0.51)	2.59 (0.50)	2.03 (0.63)
Breathing skills	1.80 (0.79)	2.70 (0.48)	0.67 (0.55)	2.43 (0.50)	0.95 (0.78)	2.50 (0.51)	2.53 (0.51)	2.24 (0.50)
Bleeding management	1.50 (1.08)	2.50 (0.71)	0.73 (0.74)	2.50 (0.51)	0.93 (0.89)	2.50 (0.55)	2.53 (0.56)	2.47 (0.56)
Immobilization	1.20 (0.63)	2.60 (0.52)	0.83 (0.75)	2.63 (0.49)	0.93 (0.73)	2.63 (0.49)	2.65 (0.49)	2.35 (0.54)

Values are presented as mean (standard deviation). Likert scale responses ranged from 0 to 4, with higher values indicating greater confidence or skill. One-year follow-up data were available for 33 participants (24 medical students and 9 practicing providers). Results are presented descriptively.

In the post-course Practical Case Scenarios (PraCS) assessments, 29/40 total participants correctly performed all the critical airway, breathing, circulation, disability, exposure, and medication interventions. The only significant difference in performance between the medical student cohort and 14 Military Hospital cohort (9 nurses and 1 physician) were the critical circulation interventions in which 19/30 medical students completed compared to 2/10 hospital cohort members (*p* = 0.032). Please refer to Table 5 for a full analysis of the PraCS performance breakdown.

**Table 5:** Post-course practical case scenario (PraCS) performance by cohort.

	Total (*N* = 40)			Medical Student Cohort (*N* = 30)			Military Staff Cohort (*N* = 10)			*p*-value*
**PraCS component**	Not performed	Some	Performed	Not performed	Some	Performed	Not performed	Some	Performed	
Critical Airway Interventions	3	10	27	2	9	19	1	1	8	0.489
Critical Breathing Interventions	3	12	25	2	8	20	1	4	5	0.522
Critical Circulation Interventions	1	18	21	1	10	19	0	8	2	0.032
Critical Disability Interventions	3	13	24	2	9	19	1	4	5	0.633
Critical Exposure Interventions	2	14	24	1	9	20	1	5	4	0.223
Critical Medications Given	0	8	32	0	5	25	0	3	7	0.388
SBAR Complete	4	0	36	2	0	28	2	0	8	0.256
Performed all components	0	0	29	7	0	23	4	0	6	0.307

*P-values calculated using Fisher’s exact test.

Course evaluations were highly positive across all assessed domains, including content appropriateness, educational materials, lectures, case scenarios, instructor effectiveness, and likelihood of recommending the course. Mean ratings ranged from 3.7 to 5.0 on a 5-point Likert scale, with no significant differences between medical students and practicing providers (all *p* > 0.05).

## Discussion

This study found that the WHO Basic Emergency Care (BEC) course was associated with significant improvements in emergency care knowledge and self-reported confidence among frontline healthcare providers in Liberia, with sustained knowledge retention at one-year follow-up. These findings are consistent with prior evaluations demonstrating short-term knowledge and confidence gains following structured emergency care education in low- and middle-income countries [1,6]. Importantly, this study extends prior BEC evaluations by assessing knowledge retention at one year following training, whereas previous evaluations have primarily focused on immediate or short-term educational outcomes. Knowledge scores remained above baseline at one year despite minimal study contact and no additional BEC instruction or refresher training during the follow-up period [7].

Sustained improvements from the pre-BEC baseline to the post-BEC and one-year timepoints support the premise that syndromic, systems-based emergency care training can promote long-term retention of foundational principles among non-specialist providers who deliver much of the acute care in resource-limited settings [2,4]. Improvements in confidence across core domains—including shock, airway management, and pediatric emergencies—align with prior BEC implementations and reinforce the role of emergency care education in strengthening health system capacity [5,6]. These findings support consideration of BEC training within undergraduate medical education and continuing professional development for frontline healthcare providers in Liberia and may inform implementation of similar standardized emergency care training programs in other resource-limited African settings.

This study represents the first pilot implementation of the WHO Basic Emergency Care (BEC) PraCS curriculum. Although knowledge and confidence remained high, only 72.5% of participants completed all PraCS components, suggesting that practical skills acquisition may require ongoing reinforcement. Future work should build on these findings by comparing outcomes across different provider groups and exploring targeted interventions to improve longitudinal engagement and skill retention.

Modest declines in airway and pharmacology knowledge at one year relative to immediate post-course assessments are consistent with known patterns of knowledge decay and suggest a potential role for targeted refresher training or continuing medical education focused on practical case scenarios and clinical skills as these were the weakest areas exhibited in the post-BEC PraCS testing [5,7].

Differences in selected practical skills and knowledge retention between provider groups likely reflect baseline training variation, underscoring the importance of adapting emergency care curricula to diverse cadres [4].

## Limitations

This quasi-experimental study lacked a control group, limiting causal inference. The single-country setting and small sample size, including only 40 participants from two healthcare provider cohorts, limit the precision and generalizability of our findings. However, the study included the entire graduating medical student class at A.M. Dogliotti School of Medicine and provides longitudinal within-participant assessment following BEC training. Confidence measures were self-reported and subject to response bias. Practical skills were assessed only immediately post-course, precluding long-term skills retention analysis. Because the one-year assessment was administered remotely, use of external resources or consultation with others could not be directly monitored and may have influenced assessment performance. Although all participants completing one-year follow-up remained in clinical practice, individual employment sites and clinical exposure during the follow-up period were not systematically collected, limiting our ability to assess how differences in clinical experience may have influenced knowledge retention. Finally, this study evaluates educational outcomes rather than patient outcomes, a limitation noted in prior emergency care education research [2,7].

## Conclusion

The WHO Basic Emergency Care course was associated with improvements in emergency care knowledge and confidence among frontline healthcare providers in Liberia, with knowledge remaining above baseline at one-year follow-up. These findings support wider implementation of WHO Basic Emergency Care training across similar African health systems and suggest that structured emergency care education can provide durable educational benefits in resource-limited settings. Future research should evaluate the impact of BEC training on patient-level outcomes, assess long-term retention of practical clinical skills, and determine the optimal frequency and format of refresher training to sustain knowledge and skills over time [1,5,6].

## Dissemination of results

The results of this study were shared with the leadership of A.M. Dogliotti School of Medicine, 14 Military Hospital, the Liberian Ministry of Health, and the African Federation for Emergency Medicine (AFEM). Findings were also shared with the study investigators and participating institutions to inform future implementation and evaluation of the WHO Basic Emergency Care training program in Liberia.

## Author contributions: credit

**OMS:** Data curation, Formal analysis, Visualisation, Writing – original draft, Writing – review and editing. **IA:** Investigation, Project administration, Resources, Writing – review and editing. **NM:** Investigation, Project administration, Resources, Writing – review and editing. AL: Conceptualisation, Investigation, Writing – review and editing. **NF:** Investigation, Writing – review and editing. **MRD:** Investigation, Project administration, Resources, Writing – review and editing. **TFM:** Investigation, Project administration, Resources, Writing – review and editing. **MJ:** Conceptualisation, Methodology, Investigation, Supervision, Writing – review and editing. **CB:** Conceptualisation, Methodology, Investigation, Supervision, Writing – review and editing. **KE:** Conceptualisation, Methodology, Investigation, Supervision, Writing – review and editing. All authors approved the final version to be published and agreed to be accountable for all aspects of the work.

## Declaration of generative AI and AI-assisted technologies in the manuscript preparation process

During the preparation of this work, the authors used University of California, San Francisco ChatGPT (OpenAI) Enterprise to assist with language editing, improving clarity and readability, restructuring sections to align with journal formatting requirements, and refining the presentation of the manuscript. All AI-generated suggestions were critically reviewed, verified, and edited by the authors. The authors take full responsibility for the content of the published article.

## Declaration of Competing Interest

The authors declared no conflicts of interest.

## References

[1] Razzak J., Beecroft B., Brown J., Hargarten S., Anand N. (2019). Emergency care research as a global health priority: key scientific opportunities and challenges. BMJ Glob Health.

[2] Thind A., Hsia R.Y., Mabweijano J., Hicks E.R., Zakariah A., Mock C.N., Debas H.T., Donkor P., Gawande A., Jamison D.T., Kruk M.E., Mock C.N. (2015). Essential surgery: disease control priorities.

[3] Anza-Ramirez C., Miranda J.J., Armocida B., Correia J.C., Van Spall H.G.C., Beran D. (2025). Characterising acute and chronic care needs: insights from the global burden of disease study 2019. Nat Commun.

[4] Razzak J.A., Kellermann A.L. (2002). Emergency medical care in developing countries: is it worthwhile?. Bull World Health Organ.

[5] Reynolds T.A., Sawe H., Rubiano A.M., Shin S.D., Wallis L.A., Mock C.N., Jamison D.T., Gelband H., Horton S. (2017). Disease control priorities: improving health and reducing poverty.

[6] World Health Organization (2018). https://www.who.int/teams/integrated-health-services/clinical-services-and-systems/emergency-and-critical-care/bec.

[7] Epps L., Ramachandran A., Yi S., Mayah A., Burkholder T., Jaung M. (2023). Implementation and outcomes of a comprehensive emergency care curriculum at a low-resource referral hospital in Liberia: a novel approach to application of the WHO basic emergency care toolkit. PLoS One.

[8] Michaeli N., De Luca G., Gitau M. (2023). Evaluation of the World Health Organization–international committee of the red cross basic emergency care course among senior medical students in Kenya. Afr J Emerg Med.

