# Supplementary material for: WHO Basic Emergency Care (BEC) training in Liberia: pre-course, post-course, and one-year outcomes
Source: Afr J Emerg Med. 2026 Oct 22;16(4):101019. doi: 10.1016/j.afjem.2026.101019 (PMC13717013; doi:10.1016/j.afjem.2026.101019)
Supplement: Supplementary file 1 [file mmc1.docx]

|  | BEC | | | |
| --- | --- | --- | --- | --- |
|  | Day 1- Monday 3/4, Friday 3/8 | Day 2- Tuesday 3/5, Saturday 3/9 | Day 3- Wednesday 3/6, Monday 3/11 | Day 4- Thursday 3/7, Tuesday 3/12 |
| 08:30 - 8:45  (15 min) | Registration | Review & Questions | Review & Questions | Review & Questions |
| 8:45 - 9:15  (30 min) | Introduction Lecture  (Icebreaker) | DIB Lecture | Shock Lecture | Medication Jeopardy 1 hr |
| 9:30 - 10:30  (60 min) | Pre-Test and pre  (45 min) |  |  | 1 hr 40 min  Skills: Assessment (primary & secondary survey)  -PRACS Case  9:45 11:45  Post-test before lunch |
| 10:30 - 11:30  (60 min) | ABCDE Lecture | DIB Small Groups | Shock Small Groups |  |
| 11:30 - 12:15  (45 min) |  | Trauma: Primary Survey | Review Pre-test Answers |  |
| 12:15 - 13:15  (60 min) | LUNCH (& PRAYER) | | | |
| 13:15 - 14:00  (45 min) | Handover Lecture | Trauma: Secondary Survey | AMS Lecture | 1 hr  Post-Test:  (Remediation)  Workbook Check  Certificates and Closing- end by 3:30 |
| 14:00 - 15:00  (60 min) | ABCDE Small Groups | Trauma: Small Groups | AMS Small Groups |  |
| 15:00 - 15:30  (30 min) | AFTERNOON TEA (& PRAYER) | | | |
| 15:30 - 16:30  (60 min) | Skills: 1.5 hours  Airway/Breathing  1)Maneuvers/OPA/NPA  2) O2 Delivery  3) Choking  4) Needle decomp/spacer/3-side chest | Skills: 1.5 hours  Circulation  1) Exam  2) external bleeding control  3)Uterine massage/PPH  4) IV cannulation | Skills: 1.5 hr  gen neuro  Immobilization and Wound  1) C-spine/ spine imm/preg/recov pos  2) Fx imm/snake  3) General/Burn/IVF  4) binder/log roll |  |
| 16:30 - 17:15  (45 min) |  |  |  |  |
| 17:15 - 17:30 | Feedback | Feedback | Feedback | Feedback |
| 17:30 - 18:00 | Faculty Meeting | Faculty Meeting | Faculty Meeting | Faculty Meeting |
